# Interaction of Per- and Polyfluoroalkyl Substances and Allostatic Load among Adults in Various Occupations

**DOI:** 10.3390/diseases10020026

**Published:** 2022-04-29

**Authors:** Tahir Bashir, Emmanuel Obeng-Gyasi

**Affiliations:** 1 Department of Built Environment, North Carolina A & T State University, Greensboro, NC 27411, USA; tmbashir@aggies.ncat.edu; 2 Environmental Health and Disease Laboratory, North Carolina A & T State University, Greensboro, NC 27411, USA

**Keywords:** PFAS, allostatic load, occupations, chronic physiological stress

## Abstract

***Objective***: This study sought to assess the associations between occupation, serum concentrations of selected of Per- and Polyfluoroalkyl Substances (PFAS), and chronic physiological stress, as operationalized by Allostatic Load (AL), among adults aged ≥20 years. ***Methods***: To explore the interactions of occupation with PFAS levels and AL, data from the National Health and Nutrition Examination Survey (NHANES) 2007–2014 were used. We performed Poisson regression modeling to evaluate AL’s relationships with PFAS concentrations and occupations on weighted data. ***Results***: The results demonstrated that increased AL was positively associated with different occupation groups such as a) Public Administration and b) Arts, Entertainment, and Recreation (*p*-values 0.018 and 0.002, respectively), and with certain PFAS concentrations (Perfluorooctanoic acid, PFOA, *p*-value = 0.002). Finally, AL had a strong association with the interaction of some PFAS such as Perfluorobutane sulfonic acid (PFBS) and occupation (AL: PFBS: occupation, *p*-value < 0.0001), with different association measures existing across varying occupations. ***Conclusions***: Occupation and PFOA seem to be associated with AL. This suggests the need of implementing further strategies to limit the exposure to stressors and PFAS in the work environment to promote longevity among the workforce in the U.S. Finally, policymakers must do more to clearly define standards and regulations in the work environment related to PFAS exposure.

## 1. Introduction

Per- and Polyfluoroalkyl substances (PFAS) are toxic organic substances that have been used worldwide as part of commodities to produce goods for people for a long time. They have been used in non-stick cookware, water-repellent clothing, stain-resistant fabrics, and carpets [1,2]. PFAS have also been employed and used in many industries such as the aerospace, automotive, construction, electronics, and military and have been used in a variety of applications, for instance, food packaging and firefighting foams, in addition to products that resist grease, water, and oil [3].

Owing to the enormous variety of PFAS chemicals, they are unevenly distributed, with different PFAS posing differing exposure risks depending on the environment [4]; these properties mean that they are used in different applications. For this reason, different PFAS are more prevalent in certain occupations. For instance, Perfluorooctane sulfonic acid (PFOS) is a compound used in military and firefighter equipment/devices [5,6]. In a study by Mamsen et al. examining firefighting foams and hydraulic fluids that are used in this occupational environment, it was found that participants had high PFOS and Perfluorohexanesulfonic acid (PFHxS) serum concentrations among individuals who held this occupation [7,8]. Other occupations have variable PFAS exposure levels, demonstrating the variability of PFAS exposures in the workplace environment.

The occupational workers in jobs such as manufacturing and assembly line workers are at greater health risk of having higher PFAS serum levels than the general population because they are likely to be exposed to individual or complex mixtures of PFAS [5]. A study conducted in Shenzhen, China, compared occupational workers to the general population on potential health concerns from exposure to PFAS; health concerns such as cancer, inflammation, liver disease, HIV/AIDS infection, diabetes, etc., were explored. The authors found that levels six PFAS, i.e., Perfluorobutanoic acid (PFBA), Perfluorooctanoic acid (PFOA), Perfluorobutane sulfonic acid (PFBS), Perfluorohexanesulfonic acid (PFHxS), Perfluorooctane sulfonic acid (PFOS), and 6:2 chlorinated Polyfluoroalkyl ether sulfonate (6:2 Cl-PFESA) in the plasma had a geometric mean of 1770 ng/mL for occupational workers and of 22.2 ng/mL for the general population [5]. These findings confirmed that manufacturing workers and assembly lines workers are at risk of health challenges compared to the general population owing to PFAS, such as PFOA exposure [5].

Stress is an unavoidable part of human life. Allostatic load (AL) provides insight into the effects of accumulated stress. The physiological response of stress processes is to promote the adaptation of the body to changing stimuli while preserving homeostasis. Allostasis refers to these physiological effects activated to achieve “stability through change” [9]. In turn, AL reflects the cost paid by the body for continual adaptation to environmental stressors [10,11]. Thus, an elevated AL results from excessive stress or inadequacy of adaptive allostatic processes. AL is often operationalized using the AL index, which captures dysregulation across various biological systems such as the cardiovascular, metabolic, and inflammatory systems [12]. This study aimed to examine the interaction associations between the serum concentration of selected PFAS, including Perfluorohexane sulfonic acid (PFHS), Pefluorodecanoic acid (PFDE), Perfluorobutane sulfonic acid (PFBS), Perfluoroheptanoic acid (PFHP), Perflurododecanoic acid (PFDO), Perfluorononanoic acid (PFNA), Perfluoroundecanoic acid (PFUA), Perfluorooctanoic acid (PFOA), and Perfluorooctane sulfonic acid (PFOS) with AL, among workers in 22 different occupations within the NHANES. We hypothesized that (a) AL would be associated with occupation and (b) the interaction of PFAS and occupation would be associated with increased levels of AL.

## 2. Materials and Methods

### 2.1. Study Population

This study used data from NHANES 2007–2014 among U.S. adults aged 20 and older to explore the relationships between occupations and PFAS serum levels with chronic stress operationalized using the AL index. The NHANES is a surveillance of the nation’s health conducted by the Centers for Disease Control and Prevention. NHANES uses a complex, multistage, stratified study design for collecting nationally representative data from the noninstitutionalized U.S. population.

### 2.2. Allostatic Load Measurement and Definition

Informed by previous studies [13,14], AL was operationalized and measured by quantifying a cumulative and health-related stress index, which depended on physiological assessments including 10 health indicators, i.e., systolic blood pressure (SBP), diastolic blood pressure (DBP), total cholesterol (TC), high-density lipoprotein (HDL) cholesterol, glycosylated hemoglobin (HbA1c), as well as albumin (Alb), triglyceride (TG), body mass index (BMI), creatinine clearance (CLCR), and C-reactive protein (CRP). AL biomarkers were transformed into quartiles based on their distribution within the data. High risk for each marker was assigned to the top 25% in the distribution for all markers apart from albumin, creatinine clearance, and HDL cholesterol, for which the bottom 25% of the distribution were considered to have the highest risk as determined by the literature [15,16,17,18,19,20,21]. Binary indicators were assigned to individuals in the study: a value of 1 if they were in the high-risk category and a value of 0 if in the low-risk category for all markers to calculate a total AL value out of 10.

### 2.3. Analytical Procedures

Automated solid-phase extraction coupled with high-performance liquid chromatography–turbo ion spray ionization–tandem mass spectrometry was used to detect PFAS serum levels. An Agilent LC1260 (St. Clara, CA, USA) AB Sciex API 5500 (Foster City, CA, USA) platform was used in the analysis.

#### PFAS Detection Limits

The detection limits were constant for all the PFAS analytes in the dataset (0.10 ng/mL). In the NHANES, two variables’ names were provided for each of these analytes. The value “0” meant that the result was at or above the limit of detection, “1” indicated that the result was below the limit of detection. For analytes with analytic results below the lower limit of detection, an imputed fill value was placed in the analyte results field. This value was the lower limit of detection divided by a square root of 2 (LLOD/sqrt(2)), which was 0.10/√2 = 0.07. So, the LOD for each PFAS was either 0.10 or 0.07. [22,23]. Further details on analytical processes and procedures are provided in the NHANES Laboratory Procedures Manual [24].

### 2.4. Statistical Analysis

This study used basic descriptive statistics to explore the mean differences of AL and PFAS levels by occupation. We performed Pearson correlations to assess the relationships between individual serum PFAS concentrations. We also performed Poisson regression models to evaluate the interaction and association of selected PFAS and occupations with AL. Missing values were imputed using standard methods [25].

All analyses factored in the study design and weights to get representative results. Wilcoxon test was conducted on continuous variables, and Wald chi-squared test on categorical variables analysis, which revealed that the variables were not normally distributed, so they were natural log-transformed. All analyses were conducted using R software, version 4.1.2 (R Foundation for Statistical Computing, Vienna, Austria) in the RStudio platform, version 2021.9.1.372, with the release name Ghost Orchid. A *p*-value < 0.05 was considered significant for all analyses in this study.

## 3. Results

We explored the PFAS levels among the 22 different occupations. There was a statistically significant higher detection rate for PFOS for mining and armed forces workers, with means of 6.327 and 6.128, respectively, among the occupations compared to other PFAS concentration levels (Table 1 and Table 2).

Table 1 and Table 2 present the mean differences between the PFAS serum concentrations for different occupations and AL levels. The mean serum PFNA concentration was the highest among all the selected PFASs for the occupations of interest in this study. In addition, serum PFOS levels were more elevated than the other PFAS concentrations when analyzed by AL levels.

We calculated descriptive statistics to assess the presence of PFAS and then we evaluated the overall mean prevalence of AL among the participants. The corresponding means (Table 3 and Table 4) showed the serum concentrations of PFOS had the highest mean (mean = 3.138) amongst the PFAS chemical compounds, by AL levels. An AL ≥ 3 was considered high, and an AL < 3 was considered low.

Poisson regression models were used on weighted data to estimate the associations of AL and occupations and the interactions of the association between occupation and PFAS and AL levels. The results revealed that an increase of AL was associated with different occupations groups, such as a) Public Administration and b) Arts, Entertainment, and Recreation (*p*-value = 0.018 and *p*-value = 0.002, respectively), and with PFAS concentrations (PFOA *p*-value = 0.002); it was also strongly associated with the interaction of PFAS and occupation (AL-by-PFBS-by-Longest held occupation) with a *p*-value < 0.001), as well as with differences existing across occupations.

The occupation groups examined in this study had various levels of association with AL; for instance, Arts, Entertainment, and Recreation had a positive and statistically significant relationship (*p*-value = 0.002) with AL. Likewise, Educational occupations had positive relationships with AL, but they were not statistically significant (*p*-values = 0.935). Regarding socioeconomics, family income was associated with AL levels, *p*-value < 0.0001 (Table 5).

The work-related environment and types of occupations, specifically, (a) Arts, Entertainment, Recreation jobs, and (b) Public Administration occupations, were associated with levels of AL, with *p*-values = 0.002 and 0.018, respectively. Table 5 shows the relationship between AL and PFAS, occupation, and income.

**Figure 1:**
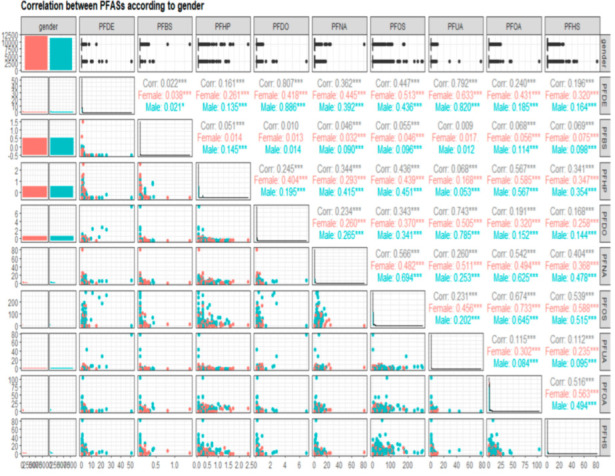
Pair plots showing correlations between selected PFAS by gender. PFAS were measured in micrograms per liter (μg/L).

Figure 1 presents a matrix of pair plots with relationships between selected PFAS. The upper triangle contains the Pearson correlations as *r*-values, which represent the strength of the association between the variables. The *r*-value can either be positive or negative depending on the direction of correlation. If the *r*-value is between 0–0.19, the correlation is considered very weak. An *r*-value between 0.2–0.39 indicates a weak correlation, an *r*-value between 0.40–0.59 a moderate one, An *r*-value between 0.6–0.79 a strong one, and an *r*-value between 0.8–1 a very strong correlation [26]. On the other hand, the correlation coefficients values (*r*-values) had the corresponding statistically significance levels (if no star is present, the variable was not statistically significant, while one (*), two (**) and three stars (***) mean that the corresponding variable was significant at 10%, 5%, and 1% levels, respectively). The graphs for continuous variables (PFAS variables considered as continuous variables) are presented as scatterplots, and the graphs for the categorical variable (gender) are presented as boxplots, with all the dots distant from the majority of points in the graphs considered as outliers.

As Figure 1 demonstrates, the correlations between selected PFAS were somewhat different. For instance, PFDO had a weak positive relationship and was not correlated with PFBS (*r* < 0.001). The aforementioned relationship was slightly different in females (*r* = 0.013) as compared with males (*r* = 0.014).

Regarding PFUA and PFDE, there was a strong and positive correlation between PFUA and PFDE (*r* = 0.792), with a moderate correlation in females (*r* = 0.431) and a very weak correlation in males (*r* = 0.185).

As shown in Figure 1, all relationships between selected PFAS were statistically significant (three stars ***), except for the correlation between PFDO and PFBS, which was not statistically significant (no stars), as mentioned above.

Table 6 shows the linear relationships between individual PFAS. PFDO and PFBS showed a weak positive correlation (*r* = 0.010). In addition, there existed a strong correlation between PFDO with PFUA, with an *r*-value = 0.743, and another strong correlation between PFDO and PFDE, with an *r*-value = 0.807. All correlations between PFAS were positive.

The Poisson regression model was used to evaluate the association of the interaction between AL and individual PFAS serum levels (AL with PFAS). There were significant associations of the interactions between AL and PFBS, *p*-value < 0.0001, AL and PFHS, *p*-value = 0.055, AL and PFDO, *p*-value = 0.048, and AL and longest occupation held, *p*-value = 0.023. On the other hand, there were no significant associations of the interactions between AL and some PFAS, such as AL and PFDE. *p*-value = 0.119, AL and PFHP, *p*-value = 0.441, AL and PFNA, *p*-value = 0.129, AL and PFOS, *p*-value = 0.155, AL and PFUA, *p*-value = 0.199, and AL and PFOA, *p*-value = 0.922, (Table 7).

For interactions of AL with PFAS concentrations and occupation (three interactions simultaneously), there were statistically significant interactions of AL with PFAS and occupations as follows: for AL with PFBS and occupation, with a *p*-value of <0.0001, and for AL with PFDO and occupation, with a *p*-value of 0.0387. On the other hand, there were no statistically significant associations of AL and PFDE with occupation, *p*-value = 0.4224, between AL, PFHP, and occupation, *p*-value = 0.890, between AL, PFNA, and occupation, *p*-value = 0.2073, between AL, PFOS, and occupation, *p*-value = 0.2414, between AL, PFUA, and occupation, *p*-value = 0.0953, between AL, PFOA, and occupation, *p*-value = 0.1525, and between AL, PFHS, and occupation, *p*-value = 0.1828, (Table 7).

**Figure 2:**
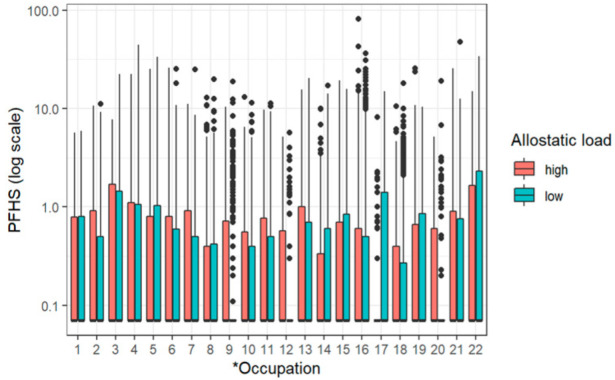
Boxplot showing the interaction between PFHS, occupation, and AL. *Occupation numbers are: 1 Agriculture, Forestry, Fishing, 2 Mining, 3 Utilities, 4 Construction, 5 Manufacturing: Durable Goods, 6 Manufacturing: Non-Durable Goods, 7 Wholesale Trade, 8 Retail Trade, 9 Transportation, Warehousing, 10 Information, 11 Finance, Insurance, 12 Real Estate, Rental, Leasing, 13 Professional, Technical Services, 14 Management, Business, Cleaning/Waste Services, 15 Education Services, 16 Health Care, Social Assistance, 17 Arts, Entertainment, Recreation, 18 Accommodation, Food Services, 19 Other Services, 20 Private Households, 21 Public Administration, and 22 Armed Forces.

Figure 2 shows the PFHS serum concentration in relation to the longest occupation held by the participants and the AL levels. PFHS was the most detected PFAS in this study, which promoted further analysis by AL and occupation. The results indicated that the highest AL was among those in the armed forces followed by utility workers.

## 4. Discussion

PFAS and occupation were shown to be associated with chronic physiological stress (AL). The mean PFAS serum concentrations for different occupations and AL levels showed significant differences in our study. The overall mean for PFNA serum concentrations was the highest among all the PFAS examined in this study. This is consistent with and very similar to a study by Graber et al. [21]. Serum PFOS levels were also more elevated than the other PFAS concentrations when explored by AL levels. This is relevant when considering the exposure risk to PFAS and how PFAS levels in various occupational settings can potentially contribute to AL among adults.

Many studies have found numerous stressors in the work environment [27,28,29]. These findings are consistent with these observations. Our study uniquely explored the role that PFAS may have on the biological response to stress in various occupations and found significant associations for PFBS and PFDO. A study by Piolanti et al. found that, in general, occupational factors were associated with chronic physiological stress, with distress associated with the increasing AL levels [30]. This association with AL levels is connected to the early course of physiological dysfunction [31], making it critical to explore these relationships in the work environment.

Our study found associations between AL and family income. The participants with higher annual income were less ‘biologically’ stressed (lower AL) than lower-income participants. For example, the association between AL and income when considering the $75,000 to $99,999 income range had a *p*-value = 0.875. On the other hand, AL was associated with income for those earning between $10,000 and $14,999, with *p*-value = 0.0225. This is in agreement with the works of Guidi et al. and Buschmann et al., who found that family income was associated with AL [32,33].

The association between AL and gender was significant; this was similar to the findings of Juster et al. and of others [34,35], which indicated that males have higher AL levels than females.

This study found that the interaction of PFAS with occupation may explain the biological response to stress. This is critical, as AL is potentially a mediator of several chronic diseases like cardiovascular disease and cancer. Therefore, it is crucial to understand how exposure to multiple PFAS in various occupations with different exposure levels affects AL. In this regard, occupational conditions are changeable and manageable factors that can be mitigated to limit their effects on AL [36]. Limiting PFAS exposure and changing conditions in the work environment can limit the exposure risk and subsequent disease.

After modifying the model (Poisson Regression) from interactions between AL and PFAS individually to interactions that included the occupations with AL and PFAS together, the statistical significance changed for some of the variables, from statistically significant to not statistically significant and vice versa. For instance, the interaction between AL and PFHS changed from significant (*p*-value = 0.05) to non-significant (*p*-value = 0.183) for these two variables. When the interaction was between three variables (AL with PFHS and occupation), the relationship remained the same for most of the variables before and after the two types of interaction tests (two variables or three variables). Nevertheless, a statistically significant and positive association between AL and occupation was observed (*p*-value = 0.023).

The study has some limitations. Firstly, the study design was cross-sectional, so we could not determine temporality. Second, the study lacks an in-depth assessment of PFAS exposure sources; for example, it is unclear whether participants were exposed to PFAS in the work environment or somewhere else. This indicates the need for a further exposure assessment to determine where individuals are being exposed to PFAS.

Nonetheless, this study has several strengths. To our knowledge, it is the first study to examine the impacts of interactions of occupational factors and PFAS on AL for people aged 20 years and older. The findings are beneficial for understanding and explaining the environmental basics of work-related stressors. This has not been extensively studied but includes critical factors that must be considered.

## 5. Conclusions

The findings in this study indicated that occupation and some PFAS are associated with AL. The increase of AL was positively associated with different occupations, for example, Public Administration, Arts, Entertainment, and Recreation jobs. AL was also associated with PFAS concentrations and with the interaction of PFAS and occupation (AL with PFAS and occupation).

We recommend that further investigation of the possible health impacts of PFAS exposure on workers outside the U.S. be performed to see how differential exposure levels to PFAS in various occupations may affect AL in other environments.

## Figures and Tables

**Table 1:** Summary mean differences in participants’ serum PFAS (PFDE, PFBS, PFHP, PFDO, PFNA) concentrations (ng/mL) according to occupational factors.

	PFAS									
	PFDE N = 23,119		PFBS N = 19,010		PFHP N = 22,250		PFDO N = 20,502		PFNA N = 23,008	
Variable	Mean	SE	Mean	SE	Mean	SE	Mean	SE	Mean	SE
* Occupations										
1	0.165	0.018	0.070	<0.001	0.087	0.005	0.078	0.002	0.450	0.070
2	0.208	0.039	0.074	0.004	0.099	0.012	0.079	0.004	0.888	0.437
3	0.133	0.013	0.070	<0.001	0.097	0.006	0.082	0.004	0.381	0.055
4	0.158	0.012	0.071	0.001	0.090	0.003	0.079	0.002	0.468	0.063
5	0.174	0.020	0.070	<0.001	0.092	0.003	0.082	0.003	0.432	0.031
6	0.170	0.020	0.070	<0.001	0.096	0.005	0.081	0.003	0.461	0.047
7	0.144	0.019	0.070	<0.001	0.086	0.003	0.077	0.002	0.378	0.060
8	0.144	0.007	0.070	<0.001	0.095	0.004	0.079	0.003	0.431	0.033
9	0.166	0.016	0.070	<0.001	0.102	0.010	0.077	0.001	0.537	0.075
10	0.136	0.011	0.070	<0.001	0.095	0.006	0.076	0.001	0.377	0.043
11	0.143	0.009	0.070	<0.001	0.089	0.003	0.076	0.001	0.371	0.026
12	0.154	0.021	0.070	<0.001	0.093	0.010	0.078	0.003	0.453	0.103
13	0.140	0.010	0.070	<0.001	0.094	0.005	0.078	0.002	0.425	0.047
14	0.139	0.010	0.070	<0.001	0.091	0.004	0.077	0.001	0.396	0.039
15	0.165	0.010	0.071	0.001	0.100	0.003	0.079	0.001	0.507	0.046
16	0.148	0.004	0.070	<0.001	0.090	0.001	0.079	0.001	0.414	0.017
17	0.154	0.019	0.074	0.004	0.104	0.011	0.080	0.002	0.488	0.090
18	0.139	0.009	0.070	<0.001	0.094	0.004	0.079	0.001	0.356	0.023
19	0.133	0.010	0.070	<0.001	0.090	0.004	0.077	0.001	0.376	0.035
20	0.181	0.042	0.070	<0.001	0.103	0.012	0.087	0.007	0.438	0.112
21	0.162	0.013	0.070	<0.001	0.088	0.004	0.080	0.002	0.443	0.030
22	0.196	0.021	0.070	<0.001	0.096	0.007	0.079	0.002	0.652	0.082

* Note: Occupations numbers are: 1 Agriculture, Forestry, Fishing, 2 Mining, 3 Utilities, 4 Construction, 5 Manufacturing: Durable Goods, 6 Manufacturing: Non-Durable Goods, 7 Wholesale Trade, 8 Retail Trade, 9 Transportation, Warehousing, 10 Information, 11 Finance, Insurance, 12 Real Estate, Rental, Leasing, 13 Professional, Technical Services, 14 Management, Business, Cleaning/Waste Services, 15 Education Services, 16 Health Care, Social Assistance, 17 Arts, Entertainment, Recreation, 18 Accommodation, Food Services, 19 Other Services, 20 Private Households, 21 Public Administration, and 22 Armed Forces.

**Table 2:** Summary mean differences in participants’ serum PFAS (PFOS, PFUA, PFOA, PFHS) concentrations (ng/mL) according to occupational factors.

	PFAS							
	PFOS N = 23,400		PFUA N = 22,907		PFOA N = 21,985		PFHS N = 23,006	
Variable	Mean	SE	Mean	SE	Mean	SE	Mean	SE
Occupations								
1	4.005	0.002	0.127	0.012	0.787	0.097	0.586	0.048
2	6.327	0.004	0.166	0.047	1.264	0.537	0.776	0.230
3	4.611	0.004	0.102	0.009	0.966	0.145	1.147	0.354
4	3.609	0.002	0.123	0.010	0.917	0.106	0.866	0.083
5	4.124	0.003	0.149	0.028	1.107	0.105	0.940	0.094
6	3.616	0.003	0.147	0.026	0.885	0.080	0.794	0.090
7	2.623	0.002	0.110	0.013	0.728	0.098	0.660	0.089
8	2.750	0.003	0.108	0.004	0.857	0.083	0.585	0.040
9	3.514	0.001	0.120	0.009	1.077	0.147	0.815	0.080
10	2.469	0.001	0.118	0.011	0.866	0.133	0.681	0.108
11	2.560	0.001	0.114	0.006	0.775	0.094	0.758	0.090
12	2.826	0.003	0.130	0.018	0.776	0.192	0.640	0.160
13	2.893	0.002	0.120	0.007	0.913	0.134	0.876	0.137
14	2.728	0.001	0.114	0.008	0.789	0.091	0.680	0.079
15	3.786	0.001	0.129	0.007	1.084	0.109	0.805	0.076
16	2.867	0.001	0.118	0.003	0.869	0.034	0.707	0.031
17	4.053	0.002	0.140	0.023	1.007	0.158	0.947	0.151
18	2.454	0.001	0.109	0.006	0.769	0.059	0.595	0.063
19	3.371	0.001	0.111	0.008	0.879	0.102	0.721	0.076
20	2.662	0.007	0.141	0.027	0.717	0.142	0.576	0.117
21	3.222	0.002	0.220	0.089	0.895	0.103	1.023	0.218
22	6.281	0.002	0.150	0.020	1.570	0.225	1.682	0.236

**Table 3:** Summary differences in participants’ serum PFAS (PFDE, PFBS, PFHP, PFDO, PFNA) concentrations (ng/mL) by Allostatic Load.

	PFAS									
	PFDE		PFBS		PFHP		PFDO		PFNA	
Variable	Mean	SE	Mean	SE	Mean	SE	Mean	SE	Mean	SE
Allostatic Load										
Low	0.146	0.003	0.070	0.000	0.090	0.001	0.079	0.001	0.425	0.020
High	0.154	0.004	0.070	0.000	0.093	0.002	0.079	0.001	0.428	0.016

**Table 4:** Summary differences in participants’ serum PFAS (PFOS, PFUA, PFOA, PFHS) concentrations (ng/mL) by Allostatic Load.

	PFAS							
	PFOS		PFUA		PFOA		PFHS	
Variable	Mean	SE	Mean	SE	Mean	SE	Mean	SE
Allostatic Load								
Low	3.010	0.173	0.119	0.005	0.835	0.030	0.733	0.029
High	3.138	0.153	0.125	0.005	0.929	0.035	0.751	0.032

**Table 5:** Poisson models used to assess the association of AL with PFAS, occupation, and annual family income.

PFAS	Coeff	95% CI	*p*-Value
PFDE	−0.0083	(−0.291, 1.010)	0.427
PFBS	0.3188	(−0.012, 0.337)	0.280
PFHP	−0.0432	(−4.823, 1.298)	0.513
PFDO	0.0639	(−0.362, 0.891)	0.444
PFNA	0.0115	(−2.421, 0.520)	0.022
PFOS	0.0006	(0.921, 5.530)	0.428
PFUA	−0.0146	(−0.156, 0.554)	0.109
PFOA	−0.0128	(−0.005, 1.833)	0.001
PFHS	0.0009	(−0.025, 1.052)	0.641
Family income	−0.0006	(0.001, 0.006)	<0.0001
* Occupations			
1	0.0525	(−0.486, 0.116)	0.215
2	0.0620	(−0.767, 0.235)	0.225
3	−0.0230	(−0.556, 0.467)	0.640
4	0.0502	(−0.496, 0.114)	0.118
5	0.0338	(−0.37, 0.190)	0.331
6	0.0451	(−0.544, 0.148)	0.226
7	0.0530	(−0.552, 0.211)	0.205
8	−0.0332	(−0.065, 0.435)	0.294
9	0.0739	(−0.498, 0.234)	0.049
10	−0.0334	(−0.158, 0.604)	0.439
11	0.0310	(−0.398, 0.249)	0.418
12	0.0308	(−0.533, 0.474)	0.579
13	−0.0100	(−0.215, 0.522)	0.794
14	0.0267	(−0.272, 0.319)	0.438
15	−0.0013	(−0.343, 0.241)	0.935
16	−0.0283	(−0.087, 0.370)	0.260
17	−0.1148	(0.223, 0.973)	0.002
18	−0.0214	(−0.242, 0.400)	0.527
19	−0.0095	(−0.332, 0.336)	0.784
20	0.0439	(−0.389, 0.468)	0.305
21	0.0841	(−0.726, −0.085)	0.018
22	0.0094	(−0.475, 0.337)	0.865

* Note: Occupations numbers are:1 Agriculture, Forestry, Fishing, 2 Mining, 3 Utilities, 4 Construction, 5 Manufacturing: Durable Goods, 6 Manufacturing: Non-Durable Goods, 7 Wholesale Trade, 8 Retail Trade, 9 Transportation, Warehousing, 10 Information, 11 Finance, Insurance, 12 Real Estate, Rental, Leasing, 13 Professional, Technical Services, 14 Management, Business, Cleaning/Waste Services, 15 Education Services, 16 Health Care, Social Assistance, 17 Arts, Entertainment, Recreation, 18 Accommodation, Food Services, 19 Other Services, 20 Private Households, 21 Public Administration, and 22 Armed Forces.

**Table 6:** Pearson correlation was used to describe the relationships between selected PFAS.

	PFHS	PFDE	PFBS	PFHP	PFDO	PFNA	PFUA	PFOA	PFOS
PFHS	1.000	0.196	0.069	0.341	0.168	0.404	0.112	0.516	0.539
PFDE	0.196	1.000	0.022	0.161	0.807	0.362	0.792	0.240	0.447
PFBS	0.069	0.022	1.000	0.051	0.010	0.046	0.009	0.068	0.055
PFHP	0.341	0.161	0.051	1.000	0.245	0.344	0.068	0.567	0.436
PFDO	0.168	0.807	0.010	0.245	1.000	0.234	0.743	0.191	0.343
PFNA	0.404	0.362	0.046	0.344	0.234	1.000	0.260	0.542	0.566
PFUA	0.112	0.792	0.009	0.068	0.743	0.260	1.000	0.115	0.231
PFOA	0.516	0.240	0.068	0.567	0.191	0.542	0.115	1.000	0.674
PFOS	0.539	0.447	0.055	0.436	0.343	0.566	0.231	0.674	1.000

**Table 7:** Poisson regression model to assess the interactions of occupation with PFAS and AL.

PFAS	Coeff	95% CI	*p*-Value
PFDE	−0.012	(−0.593, 0.557)	0.102
PFBS	−14.586	(−22.159, 0.100)	<0.0001
PFHP	0.011	(−1.452, 0.552)	0.283
PFDO	−0.452	(−2.742, 0.132)	0.054
PFNA	0.023	(−0.108, 1.072)	0.144
PFOS	−0.003	(−0.009, 5.004)	0.138
PFUA	−0.024	(−0.343, 0.413)	0.245
PFOA	0.003	(−0.055, 0.162)	0.561
PFHS	0.004	(−0.055, 0.951)	0.037
Occupation	−0.068	(−0.028, 0.0154)	0.022
^1^ AL: PFDE	0.006	(−0.713, 0.848)	0.119
AL: PFBS	3.724	(−8.595, 6.708)	<0.0001
AL: PFHP	−0.012	(−1.998, 2.085)	0.441
AL: PFDO	0.148	(−3.451, 4.131)	0.048
AL: PFNA	−0.005	(−0.176, 0.233)	0.129
AL: PFOS	0.0005	(−0.030, 0.201)	0.155
AL: PFUA	0.0006	(−0.600, 0.449)	0.199
AL: PFOA	−0.001	(−0.008, 0.091)	0.922
AL: PFHS	−0.001	(−0.007, 0.061)	0.055
AL: Occupation	0.018	(0.060, 0.065)	0.023
^2^ AL: PFDE: Occupation	−0.001	(−0.040, 0.032)	0.4224
AL: PFBS: Occupation	−0.237	(−0.905, 0.769)	<0.0001
AL: PFHP: Occupation	0.0006	(−0.128, 0.112)	0.890
AL: PFDO: Occupation	−0.007	(−0.228, 0.186)	0.0387
AL: PFNA: Occupation	0.0003	(−0.002, 0.042)	0.2073
AL: PFOS: Occupation	−0.00006	(−0.011, 0.021)	0.2414
AL: PFUA: Occupation	0.0008	(−0.001, 0.012)	0.0953
AL: PFOA: Occupation	0.0003	(−0.083, 0.503)	0.1525
AL: PFHS: Occupation	0.00001	(−0.021, 0.104)	0.1828

^1^ Interaction of AL with occupation and PFAS individually. ^2^ Interactions between three variables (AL, occupation, and PFAS) with each other.

## Data Availability

The NHANES dataset is publicly available online, accessible at cdc.gov/nchs/nhanes/index.htm (accessed on 12 February 2022).
